# *LMNA* Co-Regulated Gene Expression as a Suitable Readout after Precise Gene Correction

**DOI:** 10.3390/ijms232415525

**Published:** 2022-12-08

**Authors:** Haicui Wang, Anne Krause, Helena Escobar, Stefanie Müthel, Eric Metzler, Simone Spuler

**Affiliations:** 1 Muscle Research Unit, Experimental and Clinical Research Center, a Cooperation between the Max Delbrück Center for Molecular Medicine in the Helmholtz Association and the Charité—Universitätsmedizin Berlin, 13125 Berlin, Germany; 2 Max Delbrück Center for Molecular Medicine in the Helmholtz Association (MDC), 13125 Berlin, Germany; 3 Berlin Institute of Health, Charité Universitätsmedizin Berlin, 13125 Berlin, Germany

**Keywords:** laminopathy, muscular dystrophy, *LMNA* co-regulated genes, near-PAMless cytosine base editor, serum-induced differentiation (SID), patient-derived induced pluripotent stem cells (iPSCs)

## Abstract

*LMNA*-related muscular dystrophy is an autosomal-dominant progressive disorder caused by mutations in *LMNA*. *LMNA* missense mutations are becoming correctable with CRISPR/Cas9-derived tools. Evaluating the functional recovery of *LMNA* after gene editing bears challenges as there is no reported direct loss of function of lamin A/C proteins in patient-derived cells. The proteins encoded by *LMNA* are lamins A/C, important ubiquitous nuclear envelope proteins but absent in pluripotent stem cells. We induced lamin A/C expression in induced pluripotent stem cells (iPSCs) of two patients with *LMNA*-related muscular dystrophy, NM_170707.4 (*LMNA*): c.1366A > G, p.(Asn456Asp) and c.1494G > T, p.(Trp498Cys), using a short three-day, serum-induced differentiation protocol and analyzed expression profiles of co-regulated genes, examples being *COL1A2* and *S100A6*. We then performed precise gene editing of *LMNA* c.1366A > G using the near-PAMless (PAM: protospacer-adjacent motif) cytosine base editor. We show that the mutation can be repaired to 100% efficiency in individual iPSC clones. The fast differentiation protocol provided a functional readout and demonstrated increased lamin A/C expression as well as normalized expression of co-regulated genes. Collectively, our findings demonstrate the power of CRISPR/Cas9-mediated gene correction and effective outcome measures in a disease with, so far, little perspective on therapies.

## 1. Introduction

Laminopathies comprise a set of rare diseases genetically caused by mutations occurring in genes coding for nuclear lamina. Muscular dystrophy, metabolic, neuropathic, and premature aging disorders are within the spectrum of disorders caused by mutations in *LMNA* (OMIM *150330). Classical laminopathy refers to diseases caused by mutations in *LMNA* coding for lamin A/C, key components forming the intermediate filaments of the nuclear lamina [1].

**Figure 1:**
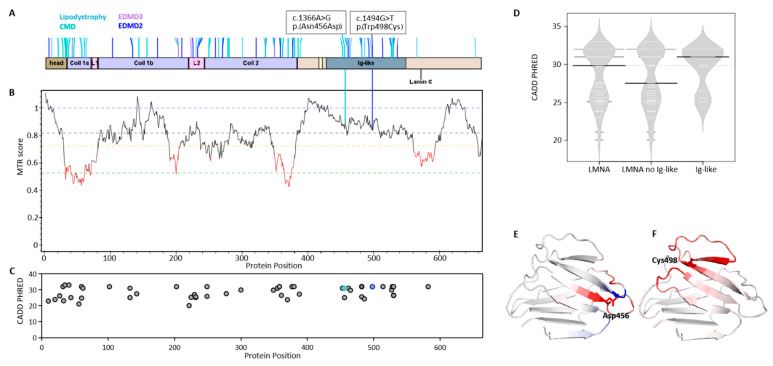
Mutation impact on lamin proteins in *LMNA*-related muscular dystrophy. (**A**) Domain organization of lamin A and lamin C with reported *LMNA*-related muscular dystrophy mutations. All reported *LMNA* variants in gnomAD database were cross-filtered for pathogenic and likely pathogenic variants with ClinVar database for the canonical transcript of *LMNA* (NM_170707.4) (data till August 2022). Four classes of muscular dystrophy are color-coded separately, including familial partial lipodystrophy (OMIM #15166), congenital muscular dystrophy (CMD, OMIM #613205), Emery-Dreifuss muscular dystrophy 2 autosomal dominant (EDMD2, OMIM #181350), and Emery-Dreifuss muscular dystrophy 3 autosomal recessive (EDMD3, OMIM #616516). The ClinVar and dbSNP ID for two selected variants: p.(Asn456Asp): ClinVar:66811, rs267607599; p.(Trp498Cys): ClinVar:66838, rs57730570. (**B**) Missense tolerance ratio (MTR). Horizontal lines show gene-specific MTR percentiles 5th, 25th, 50th, and neutrality (MTR = 1.0). MTR analysis took account of *LMNA* variants from all available exome and genome sequences for general classical laminopathy. (**C**) CADD score for selected missense mutations in *LMNA*-related muscular dystrophy. Highly pathogenic (score > 30) and pathogenic/probably damaging (> 20). (**D**) The CADD score summary for selected missense mutations of Figure 1C occurred across the protein (*LMNA*), no Ig-like domain regions (*LMNA* without Ig-like), and Ig-like domain. (**E**,**F**) 3D structure with mutation generated from Dynamut. Amino acids colored according to the vibrational entropy change upon mutation. Blue represents a rigidification of the structure and red a gain in flexibility.

*LMNA* encodes lamin A and lamin C via alternative splicing. Both lamin A and lamin C have one head, one central rod domain, and one tail domain (Figure 1A). The central rod domain is divided into sub-domains (coil 1a, L1, coil 1b, L2, and coil 2). The C-terminal tail domain consists of the nuclear localizing signal (NLS) and one Ig-like domain [2]. Thus far, the reported *LMNA* pathogenic and likely pathogenic variants of muscular dystrophy can occur at any domain of the lamin A/C (Figure 1A).

With the increasing number of CRISPR gene editing tools, there are numerous reports on precise genetic correction in muscular dystrophies. In particular, base editors allow precise single-nucleotide conversion for missense mutations without the requirement of double strand breaks [3,4]. The modified near-PAMless (PAM: protospacer-adjacent motif) base editors can even access a wider range of targets by removing the constraint of NGG PAM [5].

For laminopathy, gene editing was most frequently reported in Hutchinson-Gilford progeria syndrome (HGPS, OMIM #176670) caused by mutations in *LMNA*, leading to accumulation of the toxic protein progerin due to aberrant splicing, consequently leading to nuclear envelope alterations. With either CRISPR-Cas9 [6] or base editors [7], rescue of the aging phenotype was shown in mice through eliminating the progerin proteins. However, for most *LMNA*-related muscular dystrophies, the mutant lamin A/C proteins with one single amino acid change did not result in direct loss of function, although some mutant lamins were suggested to be associated with disrupted nuclear structure [8,9] or altered protein dynamics [10].

Patient-derived induced pluripotent stem cells (iPSCs) have been widely used to study muscular dystrophy. Lamin A/C was reported to be absent in iPSCs and expressed only in somatic cells [11,12]. One can observe upregulation of *LMNA* gene expression through long and slow differentiations into muscle-specific cells [11,13] or fast serum-induced early differentiation [12]. In an example study [13], co-regulated gene networks present at distinct myogenic stages were created via myogenic differentiation protocols for human iPSCs, which included *LMNA* and its co-regulated genes.

Here, we aimed to use the patient-derived iPSCs of *LMNA*-related muscular dystrophy to evaluate the functional recovery after near-PAMless base editing. The fast serum-induced differentiation method enabled a feasible functional readout of *LMNA* and its co-regulated gene expression.

## 2. Results

### 2.1. Mutation Impact on Lamin Proteins in LMNA-Related Muscular Dystrophy

We evaluated two patients with *LMNA* mutations in the Ig-like domain (Figure 1A). The disease phenotypes are quite different as one patient with mutation NM_170707.4: c.1366A > G, p.(Asn456Asp) has early onset congenital muscular dystrophy (CMD, OMIM #613205), while the other one NM_170707.4: c.1494G > T, p.(Trp498Cys) was previously reported to have late onset LGMD1B [14], which was reclassified as Emery-Dreifuss muscular dystrophy 2 autosomal dominant (EDMD2; OMIM #181350). Both mutations occurred in the conserved region of the protein suggested to be highly pathogenic (Supplementary Material, Figure S1).

We further performed evaluation of both mutations with different genetic tools. The missense tolerance ratio (MTR) [15], a measure of regional intolerance to missense variation, showed both mutations are not clustered in low-MTR regions of all so-far-reported *LMNA* variants (Figure 1B). However, another prediction tool, CADD, for scoring the deleteriousness of single-nucleotide variants [16], showed both mutations are highly pathogenic (CADD > 30) among the selected reported likely-pathogenic and pathogenic variants of *LMNA*-related muscular dystrophy (Figure 1C,D). The pathogenicity of both mutations required further experimental proof.

Structure prediction via Dynamut, a web server that assesses the impact of mutations on protein dynamics and stability, revealed that both mutants were overall destabilizing (Figure 1E,F).

### 2.2. Impaired Expression of LMNA after Serum-Induced Differentiation in Patient-Derived iPSCs

**Figure 2:**
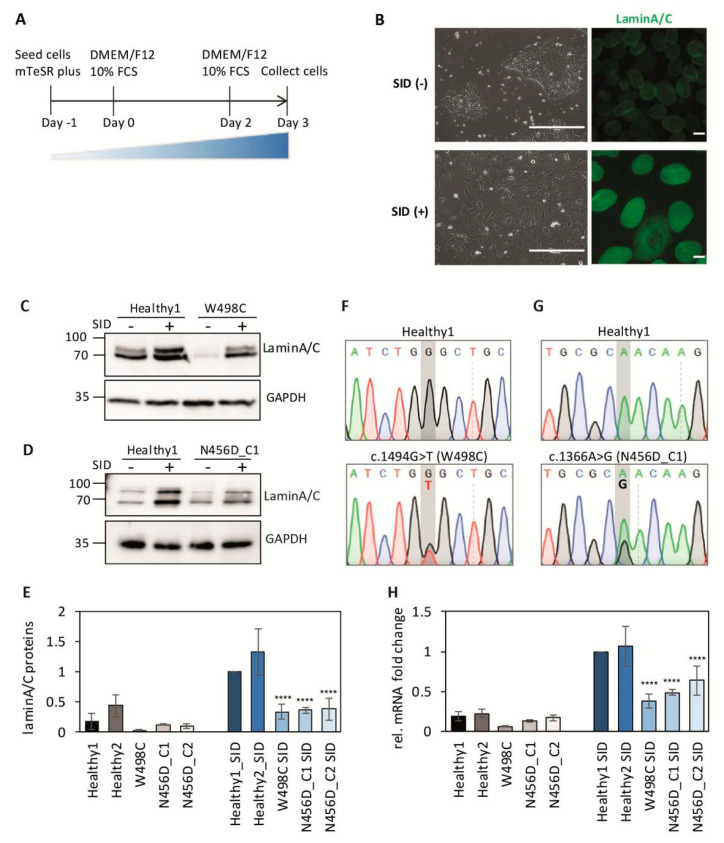
Impaired expression of *LMNA* after serum-induced differentiation in patient-derived iPSCs. (**A**) Scheme of SID protocol. (**B**) Unstained and stained iPSC *LMNA* N456D_C1 cells prior to and after SID, prior to SID as SID(−) and after SID as SID(+). Cells were stained with anti-lamin A/C antibody (green). Scale bar for unstained images 400 μm, for stained images 10 μm. (**C**–**E**) Western blot of lamin A/C protein expression in healthy and patient iPSCs prior to and after SID (N = 3; *p* < 0.0001, ****). A second healthy control iPSC and a second clone for patient iPSC *LMNA* N456D were also included (Figure S2D) for quantification, and the level of healthy1 after SID was used as reference, with grey bars as prior to SID and blue bars are SID. (**F**–**H**) *LMNA* mRNA expression in healthy and patient iPSCs prior to and after SID (N = 3; *p* < 0.0001, **** except N456D_C2). Sequencing results of reverse transcription (RT) products from mRNAs of healthy and patient iPSC prior to SID. The sequencing results after the SID can be seen in Figure S3. For quantification, the level of healthy1 after SID was used as reference with grey bars prior to SID, while blue bars are SID. Healthy1 and healthy2: two healthy controls hiPSC^LMNA_WT/LMNA_WT^; W498C: patient hiPSCs^LMNAc.1494G>T/LMNA_WT^; N456D_C1 or C2: patient hiPSCs^LMNAc.1366A>G/LMNA_WT^ clone 1 or clone 2.

To evaluate the impact of mutations on protein functions, we next generated patient-derived iPSCs from both patients hiPSCs^LMNAc.1366A>G/LMNA_WT^ and hiPSCs^LMNAc.1494G>T/LMNA_WT^ (Supplementary Material, Figure S2A,B). As *LMNA* is not expressed in iPSCs [11], a three-day serum-induced differentiation (SID) method was used for detection of *LMNA* gene expression (Figure 2A) [12]. After the three-day SID, lamin A/C was expressed on the nuclear membrane in iPSCs (Figure 2B and Supplementary Figure S2C), with significant differences between patient cells and controls after the SID (Figure 2C–E, Supplementary Material, Figure S2D). Further, mRNA expression as determined by qPCR demonstrated the same effect of SID (Figure 2F–H). In particular, we noticed that both patients have heterozygous peaks at their respective point mutation sites, suggesting transcription of both alleles, as determined by sequencing the reverse transcription products (Figure 2F,G, Supplementary Material, Figure S3), prior to or after the SID. This indicated the co–existence of both wild type and mutant lamin A/C in cell nucleus.

### 2.3. Impaired Expression of LMNA Co-Regulated Genes after SID in Patient-Derived iPSCs

Published RNA-seq data [13] indicated that *LMNA* mRNA expression rose early in development (Supplementary Material, Figure S4A) and was accompanied by a set of co-regulated genes, such as *COL1A2*, *COL4A1*, *S100A4*, *S100A6*, *S100A10* (Xi et al. [13], accessed on 2 July 2020. https://www.ncbi.nlm.nih.gov/pmc/articles/PMC7367475/bin/NIHMS1590276-supplement-Table_S6.xlsx) (Supplementary Material, Table S2 and one example *S100A6* in Figure S4B).

**Figure 3:**
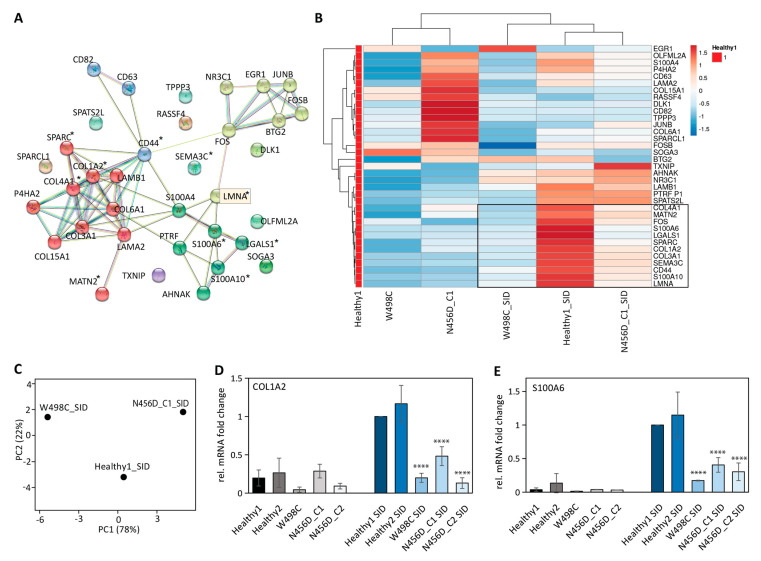
*LMNA* co-regulated genes prior to and after SID. (**A**) Interaction network of *LMNA* co-regulated genes during myogenesis (Supplementary Material, Table S2). The network was created via STRING, and proteins are clustered via MCL clustering (minimum required interaction score 0.400). Common genes with impaired expressions after SID in two patient iPSCs were marked with (*). (**B**) Heatmap of gene expression in iPSCs prior to and after SID for *LMNA* co-regulated genes. All gene expression was normalized to healthy control prior to SID. Gene expressions were clustered using correlation distance and average linkage (raw data in Table S3). Common genes with impaired expressions after SID in two patient iPSCs were marked within the black frame. (**C**) PCA analysis of gene expression file after SID of healthy and patient-derived iPSCs revealed three separated groups. (**D**,**E**) Selected gene markers among *LMNA* co-regulated genes for the next step gene editing (N = 3; *p* < 0.0001, ****). Two healthy controls (healthy1 and healthy2) and two clones from patient carrying N456D mutation (N456D_C1, N456D_C2) were included in validating the selected gene expression. Healthy1 and healthy2: hiPSC^LMNA_WT/LMNA_WT^; W498C: patient hiPSCs^LMNAc.1494G>T/LMNA_WT^; N456D_C1 or C2: patient hiPSCs^LMNAc.1366A>G/LMNA_WT^ clone 1 or clone 2.

We submitted the published *LMNA* and the set of co-regulated genes to String, a protein-protein interaction network server (https://string-db.org/). Three major clusters of proteins were identified (Figure 3A and Supplementary Figure S4C–E). One cluster was composed of extracellular matrix proteins, including several collagens and laminins. The second cluster was transcription regulators, including transcription factor complex AP-1 members Fos, FosB, and JUNB. The third cluster included calcium binding or regulating proteins (S100A4, S100A6, S100A10, AHNAK) and some metabolic-related proteins (PTRF and SOGA3).

We then investigated the relative gene expression changes in *LMNA* and its co-regulated genes after SID. Along with upregulated *LMNA* expression after SID, a proportion of co-regulated genes were also upregulated in iPSCs from the healthy donor (Figure 3B and Supplementary Material, Table S3 sample Healthy1_SID). Around half of the *LMNA* co-regulated genes gained more than three-fold changes in expression after SID, such as *COL1A2*, *SPARC*, and *S100A6*, suggesting *LMNA* and some of its co-regulated genes can also be used as differentiation markers for the SID method.

In the patient-derived iPSCs after SID, a significant impairment was observed in the expression of *LMNA* and its co-regulated genes (Figure 3B). Not surprisingly, the impaired gene expression profiles after SID were distinct between the two patients (Figure 3C), with only some of the co-regulated genes commonly impaired (as marked out with * in Figure 3A or within the black frame in Figure 3B). This indicated that mutation p.(Asn456Asp) and p.(Trp498Cys) indeed impacted the lamin A/C functions differently, consistent with the distinct disease manifestation of these two patients.

Four impaired genes (*SPARC*, *COL1A2*, *COL4A1*, *MATN2*) belonged to the extracellular matrix protein cluster, and another two impaired genes (*S100A6*, *S100A10*) belonged to the calcium binding protein cluster. The impaired expression of three selected genes, *S100A6*, *COL1A2,* and *SPARC,* after SID were validated in iPSCs of two healthy controls and three clones from two patients (Figure 3D,E, Supplementary Material, Figure S5A). *BTG2*, a low-correlated and low-fold change gene (Supplementary Material, Tables S2 and S3) as a negative control, showed only mild impairment (Supplementary Material, Figure S5B). The validated impaired genes, in particular *S100A6,* which was the highest upregulated gene after SID (see fold change in Supplementary Material, Table S3), will be further used for assessing the function of lamins.

### 2.4. Near-PAMless Cytosine Base Editing of LMNA N456D Mutation in Patient-Derived iPSCs

*LMNA* c.1366A > G can, by in silico prediction, be repaired by the cytosine base editor (CBE) to convert the G to A. However, the wild type CBE requires an efficient deamination window, typically from positions 4 to 8 within the protospacer, counting the end distal to the protospacer-adjacent motif (PAM) NGG as position 1 in the sgRNA [3]. In the case of *LMNA* c.1366A > G, there is no NGG PAM available for placing the c.1366G within the efficient deamination window.

**Figure 4:**
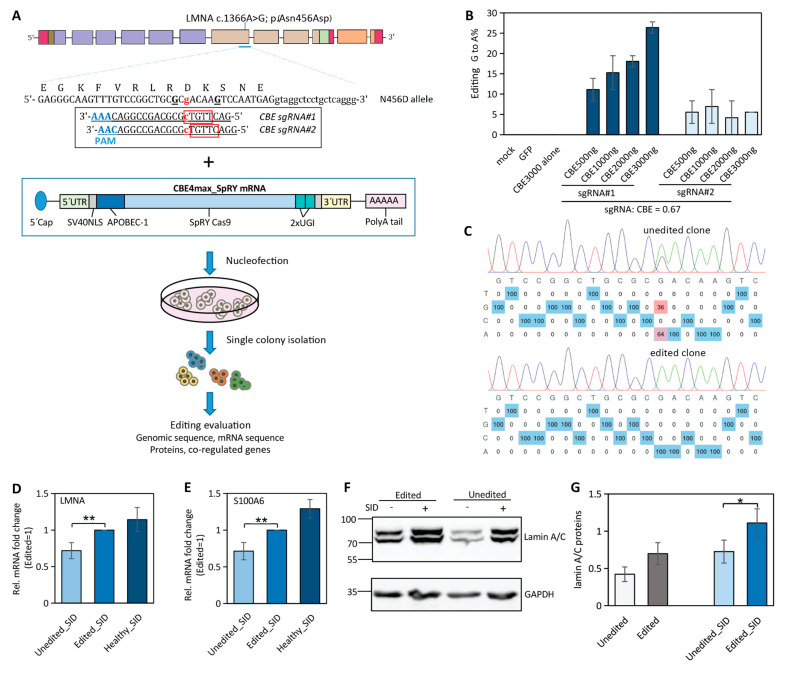
Cytosine base editing in *LMNA* patient-derived iPSCs. (**A**) Scheme of using mRNA of CEB4max_SpRY and sgRNA to correct mutation in *LMNA* mutated allele and the following evaluation procedures. Editing window in red frame; PAM sequences were highlighted in light blue. (**B**) Editing efficiency of sgRNAs in iPSC *LMNA* N456D_C2 cells. (**C**) Genome sequence of unedited and edited clones. (**D**,**E**) The mRNA levels of *LMNA* and its co-regulated gene in edited vs unedited iPSC N456D_C2 after SID (N = 3; *p* < 0.005, **). Healthy_SID includes both healthy1 and healthy2. (**F**,**G**) Lamin A/C protein levels of edited vs unedited iPSC N456D_C2 prior to and after SID (N = 3; *p* < 0.05, *). Healthy_SID includes both healthy1 and healthy2. Healthy1 and healthy2: hiPSC^LMNA_WT/LMNA_WT^; W498C: patient hiPSCs^LMNAc.1494G>T/LMNA_WT^; N456D_C1 or C2: patient hiPSCs^LMNAc.1366A>G/LMNA_WT^ clone 1 or clone 2.

The near-PAMless CBE4max_SpRY can access a wide target range with NRN PAM by removing the constraint of NGG PAM [5]. The sgRNAs were designed in a strategy not only to have the c.1366G in the efficient deamination window but also to avoid the bystander editing of the neighboring G to introduce extra disease-causing mutations (Figure 4A) as the bystander c.1364G > A was reported to cause cardiomyopathy (OMIM #115200) in the ClinVar database. The sgRNA1 placed the target c.1366G at efficient editing window position 8 (C at the reverse strand), with c.1364G at editing position 10. A second sgRNA with the c.1366G at position 9, which is slightly out of the efficient editing window, was also included to secure the c.1364G unedited.

Initially, the CBE4max_SpRY and sgRNAs were delivered to the iPSCs via a double vector system with lipofectamine, and the positive transfected cells were FACS sorted with the GFP reporter from the CBEmax_SpRY vector (Supplementary Material, Figure S6A). The test results showed higher editing efficiency with sgRNA1 compared to sgRNA2 in patient-derived iPSCs (Supplementary Material, Figure S6B). However, we observed very high cellular toxicity due to double vector transfection and quite variable editing efficiency due to the uneven delivery of the base editor vector and sgRNA vector.

To minimize the toxicity to iPSCs from the delivery of the base editor and sgRNAs, customized mRNAs for both the CBE4max_SpRY and sgRNAs were delivered to cells via nucleofection (Figure 4A). There was significantly reduced toxicity at the concentration of applied mRNAs compared to the double vector system. A high correlation between the concentration of the mRNA:sgRNA complex and the editing efficiency for sgRNA1 was observed but not for sgRNA2 as it showed poor editing efficiency (Figure 4B). Single edited and unedited iPSC clones were isolated (Figure 4C) and subjected to downstream functional evaluation.

### 2.5. Correction of LMNA Mutation in iPSCs Partially Restores LMNA and Its Co-Regulated Gene Expression

The *LMNA* gene expression at either the protein level or mRNA level was significantly lower in patient-derived iPSCs compared to the healthy ones after SID (Figure 2E,H). We found that, in the edited iPSCs from *LMNA* N456D patients, there was significantly increased expression of *LMNA* and its co-regulated gene S100A6 compared to the unedited ones, although it was still lower than in the healthy controls (Figure 4D,E). The other co-regulated gene, COL1A2, only showed very slight recovery in the edited cells after SID (Supplementary Material, Figure S7A). Consistent with the *LMNA* mRNA expression, the lamin A/C protein level was also increased in the edited iPSCs but was relatively lower than the healthy ones (Figure 4F,G, Supplementary Material, Figure S7B,C).

Our results indicated partial recovery in *LMNA,* and its co-regulated gene expression was achieved via base editing in patient-derived iPSCs.

## 3. Discussion

In autosomal-dominant diseases, gene correction needs to be allele-specific as in compound heterozygous disorders. The readout on the protein itself is compromised because of existing protein expression derived from the healthy allele. In *LMNA*-associated diseases such as muscular dystrophy, outcome measures are particularly difficult to assess because of the peculiar localization of lamin A/C at the nuclear membrane and difficulty to survive in a homozygous model. We demonstrate here an elegant approach to quickly express lamin A/C in iPSC-derived-cells in order to assess the functional impact of gene correction on a molecular level.

We made use of two previously demonstrated features of lamin A/C: (1) expression of *LMNA* mRNA in a very early development stage. Although iPSCs are lamin A/C negative, SID induces lamin A/C expression after three days. (2) Expression of genes that are co-regulated with *LMNA* and are altered in the case of *LMNA* mutations: taking advantage of indirect effects of *LMNA* mutations facilitated gene editing readout in terms of time and money.

Co-regulated genes vary between mutations of lamin A/C consistent with the varied disease severity, with a certain similarity due to mutations in one gene. The set of *LMNA* co-regulated genes are either involved in general tissue development, such as *COL1A2* and *SPARC* for osteogenesis [17], or in general cellular regulations, such as S100 calcium binding proteins for cell proliferation, differentiation, inflammation, migration, and/or invasion, apoptosis, Ca^2+^ homeostasis, and energy metabolism [18]. The recovery of their expression would be a positive indication of the overall functional recovery of cells following genetic correction.

As a proof of concept, we used the near PAMless CBE variant to repair the *LMNA* c.1366A > G mutation and performed evaluation of gene expression for *LMNA* and *S100A6*. S100A6, the S100 family member that predominantly localizes in the sarcoplasmic reticulum [19], exhibited the highest *LMNA* correlation with the myogenic differentiation protocol (Supplementary Material, Table S2) and the highest upregulation after SID (Supplementary Material, Table S3). It also showed correlated recovery along with *LMNA* following genetic correction.

Altered calcium cycling has been reported in *LMNA*-related cardiomyopathy in both an iPSC-derived disease model and mouse models [20,21]. Sarcolipin, an inhibitor of sarco/endo plasmic reticulum Ca2+-ATPase (SERCA), was significantly upregulated in different types of muscular dystrophies [22], including *LMNA*-related cardiomyopathy, while downregulation of sarcolipin led to delays in cardiac dysfunction in a mouse model [21]. On the other hand, there was also a report on the involvement of Ryanodine receptor remodeling in the same *LMNA*-related cardiomyopathy [23]. Together with the involvement of S100A6 in *LMNA*-related muscular dystrophy from our study, altered calcium cycling might be a common disturbance due to *LMNA* mutations, although the involved calcium regulators may differ in different subtypes of laminopathy.

Classical laminopathy comprises a large number of mutations in *LMNA*, with more than 300 likely pathogenic and pathogenic variants reported in the ClinVar database. Variant base editors enable high accessibility to the target region at the genomic sequence [5,24,25]. Thus, more that 40% of *LMNA* missense mutations can be potentially corrected with either cytosine base editor or adenine base editor.

For clinical translation, a stricter PAM would be required because of safety concerns and potential off-target effects. Many attempts have been made regarding improvement either via creating high fidelity versions of base editors [26] or using optimized transient approaches, such as RNP or mRNA delivery, to reduce potential off-targets from delivery of plasmids or stable integrations that provide a longer window of opportunity for off-target mutagenesis [27]. We initially observed high toxicity of delivering the vector forms of CBE and sgRNAs while obtaining significant improvement with mRNA delivery.

Patient-derived iPSCs have the characteristics of immortality, multi-lineage differentiation potential, and patient genomic specificity, making them a good choice to optimize experimental conditions for gene therapy [28]. Further, iPSCs have been widely used in studying myogenesis through myogenic differentiation methods, which, in general, takes from weeks to months [13,29,30,31]. For *LMNA*-related muscular dystrophy, it also required a long myogenic differentiation process in order to make the endpoint functional evaluations, such as the nuclei morphology changes [32]. By applying a three-day fast differentiation method SID, we can obtain a gene expression profile of *LMNA* and its co-regulated genes. This will provide a fast and feasible functional readout for high-throughput screening in precise gene correction of *LMNA* mutations.

In summary, we demonstrated application of near-PAMless base editing for an unconstrained site for precise gene correction in *LMNA* with very effective outcome measures of *LMNA* and its co-regulated genes, with fast SID in *LMNA*-related muscular dystrophy. The coupling of CRISPR/Cas9-mediated gene correction with the effective outcome measures may facilitate future gene therapy progress in *LMNA*-related muscular dystrophies and also other subtypes of laminopathy.

## 4. Materials and Methods

### 4.1. iPSC Culture and Serum-Induced Differentiation (SID)

The iPSCs were generated and characterized as described previously [33,34] from peripheral blood mononuclear cells. For SID, cells were split with 0.5 mM EDTA (Thermo Fisher Sci., Waltham, MA, USA) to iPSC aggregates with 5–8 cells and seeded the cells in 6× well plate coated with hESC-grade Matrigel (Corning, New York, NY, USA) to obtain 30–40% confluency the next day. SID medium containing the DMEM F-12 basal medium (Gibco, Thermo Fisher Scientific, Waltham, MA, USA) with 10% FBS (Sigma, St. Louis, MO, USA) was added to cells 24 h after plating the cells. Cells were maintained in SID medium for 3 days. Cell morphology during SID was monitored via EVOS cell imaging system (Thermo Fisher Sci., Waltham, MA, USA). The iPSCs without SID were seeded equally and maintained in mTeSR plus medium (Stemcell Tech, Vancouver, BC, Canada) for the same time length.

### 4.2. iPSC Nucleofection with mRNA CBE4max_SpRY and sgRNA

Codon-optimized CBE4max_SpRY mRNA from previous publication [5] was purchased from AmpTec (Hamburg, Germany). The sgRNAs were purchased from Synthego (California, CA, USA).

The iPSCs were detached with Accutase (Thermo Fisher Sci., Waltham, MA, USA). Total 300,000 cells per reaction were spun down and washed once with PBS. Cells were resuspended in 20 μL reaction with 18 μL P3 Primary Cell Nucleofector Solution (Lonza, Basel, Switzerland) premixed with 2 μL mRNA and sgRNA at desired concentration. The cells were electroporated by Amaxa 4D Nucleofector (Lonza, Basel, Switzerland) using the X Unit in 16-well nucleofection cuvettes with the program CB-150. Afterwards, 80 μL of pre-warmed mTeSR plus medium was added to each cuvette and the cells were transferred to a single well of a 6-well plate for standard iPSC culture. Fresh medium was changed regularly, and cells were analyzed 96 h after nucleofection.

### 4.3. iPSC Transfection with Double Vectors

The vector with sgRNA was described in the previous study [35] and used in this study with removal of the Cas9-T2A-Venus via restriction enzymes. The pCAG_CBE4max_SpRY was purchased from Addgene (Plasmid #139999).

The transfection was performed as previously described in Escobar et al. [35]. Briefly, the iPSCs were plated on 6-well plate coated with hESC-grade Matrigel (Corning, New York, NY, USA) at a density of 300,000 cells per well in mTeSR1 medium (Stemcell Technologies, Vancouver, BC, Canada) containing 10 μM Y-27632 2HCl (Selleckchem, Planegg, Germany). After 24 h, cells were switched to fresh mTeSR1 medium and transfected using Lipofectamine Stem Transfection Reagent (Thermo Fisher Scientific, Waltham, MA, USA) following manufacturer’s instructions. Two days after transfection, Venus+ cells were sorted using a FACSAria cell sorter (BD Biosciences, Franklin Lakes, NJ, USA) and cultured in mTeSR1 containing 10 μM Y-27632 2HCl.

### 4.4. Genotype Sequencing and Analysis of Edited Cells

The genomic DNA was extracted with Agencourt AMPure XP beads (Beckman Coulter, Indianapolis, IN, USA) according to manufacturer’s instructions. PCR fragment longer than 200 bp containing the target sequence was amplified using Q5 or Phusion High-Fidelity DNA Polymerase (New England Biolabs, Ipswich, MA, USA) and sent for Sanger sequencing. Sequence chromatograms were analyzed with EditR. Primers used for sequencing were listed in Supplementary Material Table S1.

### 4.5. RT-PCR and qPCR

Total RNA was isolated either with RNeasy Mini Kit (Qiagen, Venlo, The Netherlands) or with TRIzol through standard procedures, followed by reverse transcription via QuantiTect Reverse Transcription kit (Qiagen, Venlo, The Netherlands). RT-PCR was performed with Q5 High-Fidelity DNA polymerase (New England Biolabs, Ipswich, MA, USA). Quantitative PCR (qPCR) was performed using KAPA SYBR FAST qPCR Master Mix Universal (Sigma-Aldrich, St. Louis, MO, USA) in a CFX Connect Real-Time System (Bio-Rad, Hercules, CA, USA). Data were evaluated with the 2^−ΔΔCT^ method. GAPDH was used as reference gene. (Primers used for RT and qPCR were listed in Supplementary Material Table S1).

The heatmap of gene expression and PCA analysis results were generated with web tool ClustVis.

### 4.6. Western Blotting

Cells were lysed in lysis buffer (50 mM Tris-HCl, 150 mM NaCl, 0.5% Triton-X100, 0.5% sodium deoxycholate, 1 mM EDTA, 50 mM sodium fluoride, and 1 mM sodium orthovanadate) containing protease inhibitors for 30 min on ice. Each sample of 20 μg protein in sample buffer (350 mM Tris–HCl, 30% glycerol, 10% sodium dodecyl sulfate, 600 mM DTT, and 0.05% bromophenol blue) was boiled at 90 °C for 10 min and loaded into 8–16% gradient or 10% Tris–glycine acrylamide gel (Thermo Fisher Sci., Waltham, MA, USA). After blotting, the blot was incubated with primary anti-Lamin A/C antibody (1:1000, ab238303 Abcam) in 3% BSA/PBST at room temperature for 1 h. The HRP-conjugated secondary antibodies were incubated at room temperature for 45 min. The membrane was incubated with ECL reagent (Thermo Fisher Sci., Waltham, MA, USA) and imaged using a VWR CHEMI only system (VWR International GmbH). Quantification was performed with ImageJ (NIH).

### 4.7. Immunostaining

The iPSCs were cultured on μ-Slides (8-well, ibidi) precoated with Matrigel (Corning, New York, NY, USA)). Cells were fixed with 3.7% formaldehyde for 15 min at room temperature, permeabilized with 0.25% Triton X-100 for 10 min at room temperature, and blocked in 1% BSA/PBS for 30 min at room temperature. Samples were incubated with anti-Lamin A/C antibody (1:1000, ab238303 Abcam) in 1% BSA/PBS overnight at 4 °C. After washing, AlexaFluor 488-conjugated secondary antibodies (Thermo Fisher Sci., Waltham, MA, USA) were incubated for 1 h at room temperature. Nuclei were counterstained with Hoechst 33258 (0.5 μg/mL, Sigma-Aldrich, St. Louis, MO, USA). Samples were imaged with a Zeiss LSM 700 confocal microscope (Carl Zeiss, Jena, Germany).

### 4.8. Statistical Analysis

Statistical analysis was performed using GraphPad v9.3.1. Unpaired two-tailed *t*-test was used to compare two experimental groups. A *p* value < 0.05 was considered statistically significant.

### 4.9. Study Approval

Research use of human material was approved by the regulatory agencies (EA2/175/17, Charité Universitätsmedizin Berlin), and written informed consent was obtained from donors or legal guardians.

## Data Availability

Primary Western blot data are included in the manuscript. All other primary data are available upon science-based request from the corresponding authors.

## Supplementary Materials

The following supporting information can be downloaded at: https://www.mdpi.com/article/10.3390/ijms232415525/s1.

## Abbreviations

SID	serum-induced differentiation
iPSCs	induced pluripotent stem cells
CMD	congenital muscular dystrophy
EDMD2	Emery–Dreifuss muscular dystrophy 2
EDMD3	Emery–Dreifuss muscular dystrophy 3
MTR	missense tolerance ratio
CBE	cytosine base editor
PAM	protospacer-adjacent motif

## References

[1] Donnaloja F., Carnevali F., Jacchetti E., Raimondi M.T. (2020). Lamin A/C Mechanotransduction in Laminopathies. Cells.

[2] Ahn J., Jo I., Kang S.M., Hong S., Kim S., Jeong S., Kim Y.H., Park B.J., Ha N.C. (2019). Structural basis for lamin assembly at the molecular level. Nat. Commun..

[3] Komor A.C., Kim Y.B., Packer M.S., Zuris J.A., Liu D.R. (2016). Programmable editing of a target base in genomic DNA without double-stranded DNA cleavage. Nature.

[4] Gaudelli N.M., Komor A.C., Rees H.A., Packer M.S., Badran A.H., Bryson D.I., Liu D.R. (2017). Programmable base editing of A•T to G•C in genomic DNA without DNA cleavage. Nature.

[5] Walton R.T., Christie K.A., Whittaker M.N., Kleinstiver B.P. (2020). Unconstrained genome targeting with near-PAMless engineered CRISPR-Cas9 variants. Science.

[6] Santiago-Fernández O., Osorio F.G., Quesada V., Rodríguez F., Basso S., Maeso D., Rolas L., Barkaway A., Nourshargh S., Folgueras A.R. (2019). Development of a CRISPR/Cas9-based therapy for Hutchinson-Gilford progeria syndrome. Nat. Med..

[7] Koblan L.W., Erdos M.R., Wilson C., Cabral W.A., Levy J.M., Xiong Z.M., Tavarez U.L., Davison L.M., Gete Y.G., Mao X. (2021). In vivo base editing rescues Hutchinson-Gilford progeria syndrome in mice. Nature.

[8] Earle A.J., Kirby T.J., Fedorchak G.R., Isermann P., Patel J., Iruvanti S., Moore S.A., Bonne G., Wallrath L.L., Lammerding J. (2020). Mutant lamins cause nuclear envelope rupture and DNA damage in skeletal muscle cells. Nat. Mater..

[9] Leong E.L., Khaing N.T., Cadot B., Hong W.L., Kozlov S., Werner H., Wong E.S.M., Stewart C.L., Burke B., Lee Y.L. (2022). Nesprin-1 LINC complexes recruit microtubule cytoskeleton proteins and drive pathology in Lmna mutant striated muscle. Hum Mol Genet..

[10] Gilchrist S., Gilbert N., Perry P., Ostlund C., Worman H.J., Bickmore W.A. (2004). Altered protein dynamics of disease-associated lamin A mutants. BMC Cell Biol..

[11] Liu G.H., Barkho B.Z., Ruiz S., Diep D., Qu J., Yang S.L., Panopoulos A.D., Suzuki K., Kurian L., Walsh C. (2011). Recapitulation of premature ageing with iPSCs from Hutchinson-Gilford progeria syndrome. Nature.

[12] Bergqvist C., Jafferali M.H., Gudise S., Markus R., Hallberg E. (2017). An inner nuclear membrane protein induces rapid differentiation of human induced pluripotent stem cells. Stem Cell Res..

[13] Xi H., Langerman J., Sabri S., Chien P., Young C.S., Younesi S., Hicks M., Gonzalez K., Fujiwara W., Marzi J. (2020). A Human Skeletal Muscle Atlas Identifies the Trajectories of Stem and Progenitor Cells across Development and from Human Pluripotent Stem Cells. Cell Stem Cell..

[14] Spuler S., Geier C., Osterziel K.J., Gutberlet M., Genschel J., Lehmann T.N., Zinn-Justin S., Gilquin B., Schmidt H. (2005). A new LMNA mutation causing limb girdle muscular dystrophy 1B. J. Neurol..

[15] Traynelis J., Silk M., Wang Q., Berkovic S.F., Liu L., Ascher D.B., Balding D.J., Petrovski S. (2017). Optimizing genomic medicine in epilepsy through a gene-customized approach to missense variant interpretation. Genome Res..

[16] Rentzsch P., Schubach M., Shendure J., Kircher M. (2021). CADD-Splice—Improving genome-wide variant effect prediction using deep learning-derived splice scores. Genome Med..

[17] Etich J., Leßmeier L., Rehberg M., Sill H., Zaucke F., Netzer C., Semler O. (2020). Osteogenesis imperfecta-pathophysiology and therapeutic options. Mol. Cell Pediatr..

[18] Gonzalez L.L., Garrie K., Turner M.D. (2020). Role of S100 proteins in health and disease. Biochim. Biophys. Acta Mol. Cell Res..

[19] Mandinova A., Atar D., Schafer B.W., Spiess M., Aebi U., Heizmann C.W. (1998). Distinct subcellular localization of calcium binding S100 proteins in human smooth muscle cells and their relocation in response to rises in intracellular calcium. J. Cell Sci..

[20] Shah D., Virtanen L., Prajapati C., Kiamehr M., Gullmets J., West G., Kreutzer J., Pekkanen-Mattila M., Heliö T., Kallio P. (2019). Modeling of LMNA-Related Dilated Cardiomyopathy Using Human Induced Pluripotent Stem Cells. Cells.

[21] Morales Rodriguez B., Domínguez-Rodríguez A., Benitah J.P., Lefebvre F., Marais T., Mougenot N., Beauverger P., Bonne G., Briand V., Gómez A.M. (2020). Activation of sarcolipin expression and altered calcium cycling in LMNA cardiomyopathy. Biochem. Biophys Rep..

[22] Bal N.C., Gupta S.C., Pant M., Sopariwala D.H., Gonzalez-Escobedo G., Turner J., Gunn J.S., Pierson C.R., Harper S.Q., Rafael-Fortney J.A. (2021). Is Upregulation of Sarcolipin Beneficial or Detrimental to Muscle Function?. Front. Physiol..

[23] Dridi H., Wu W., Reiken S.R., Ofer R.M., Liu Y., Yuan Q., Sittenfeld L., Kushner J., Muchir A., Worman H.J. (2021). Ryanodine receptor remodeling in cardiomyopathy and muscular dystrophy caused by lamin A/C gene mutation. Hum. Mol. Genet..

[24] Wang Y., Gao R., Wu J., Xiong Y.C., Wei J., Zhang S., Yang B., Chen J., Yang L. (2019). Comparison of cytosine base editors and development of the BEable-GPS database for targeting pathogenic SNVs. Genome Biol..

[25] Miller S.M., Wang T., Randolph P.B., Arbab M., Shen M.W., Huang T.P., Matuszek Z., Newby G.A., Rees H.A., Liu D.R. (2020). Continuous evolution of SpCas9 variants compatible with non-G PAMs. Nat. Biotechnol..

[26] Zhang W., Yin J., Zhang-Ding Z., Xin C., Liu M., Wang Y., Ai C., Hu J. (2021). In-depth assessment of the PAM compatibility and editing activities of Cas9 variants. Nucleic Acids Res..

[27] Vicencio J., Sánchez-Bolaños C., Moreno-Sánchez I., Brena D., Vejnar C.E., Kukhtar D., Ruiz-López M., Cots-Ponjoan M., Rubio A., Melero N.R. (2022). Genome editing in animals with minimal PAM CRISPR-Cas9 enzymes. Nat. Commun..

[28] De Masi C., Spitalieri P., Murdocca M., Novelli G., Sangiuolo F. (2020). Application of CRISPR/Cas9 to human-induced pluripotent stem cells: From gene editing to drug discovery. Hum. Genom..

[29] Magli A., Perlingeiro R.R.C. (2017). Myogenic progenitor specification from pluripotent stem cells. Semin. Cell Dev. Biol..

[30] Chal J., Oginuma M., Al Tanoury Z., Gobert B., Sumara O., Hick A., Bousson F., Zidouni Y., Mursch C., Moncuquet P. (2015). Differentiation of pluripotent stem cells to muscle fiber to model Duchenne muscular dystrophy. Nat. Biotechnol..

[31] Shelton M., Metz J., Liu J., Carpenedo R.L., Demers S.P., Stanford W.L., Skerjanc I.S. (2014). Derivation and expansion of PAX7-positive muscle progenitors from human and mouse embryonic stem cells. Stem Cell. Rep..

[32] Steele-Stallard H.B., Pinton L., Sarcar S., Ozdemir T., Maffioletti S.M., Zammit P.S., Tedesco F.S. (2018). Modeling Skeletal Muscle Laminopathies Using Human Induced Pluripotent Stem Cells Carrying Pathogenic LMNA Mutations. Front Physiol..

[33] Metzler E., Telugu N., Diecke S., Spuler S., Escobar H. (2020). Generation of three age and gender matched pairs of human induced pluripotent stem cells derived from myoblasts (MDCi011-A, MDCi012-A, MDCi013-A) and from peripheral blood mononuclear cells (MDCi011-B, MDCi012-B, MDCi013-B) from the same donor. Stem Cell Res..

[34] Metzler E., Telugu N., Diecke S., Spuler S., Escobar H. (2020). Generation of two human induced pluripotent stem cell lines derived from myoblasts (MDCi014-A) and from peripheral blood mononuclear cells (MDCi014-B) from the same donor. Stem Cell Res..

[35] Escobar H., Krause A., Keiper S., Kieshauer J., Müthel S., de Paredes M.G., Metzler E., Kühn R., Heyd F., Spuler S. (2021). Base editing repairs an SGCA mutation in human primary muscle stem cells. JCI Insight.

