# Supplementary material for: LMNA Co-Regulated Gene Expression as a Suitable Readout after Precise Gene Correction
Source: Int J Mol Sci. 2022 Dec 8;23(24):15525. doi: 10.3390/ijms232415525 (PMC9778678; doi:10.3390/ijms232415525)
Supplement: Supplementary file 1 [file ijms-23-15525-s001.zip › ijms-2049023-supplementary.pdf]

LMNA co−regulated gene expression as a suitable readout after precise gene correction

Haicui Wang[1,2,3*], Anne Krause[1,2,3], Helena Escobar[1,2,3], Stefanie Müthel[1,2,3], Eric Metzler[1,2,3], Simone Spuler[1,2,3*]

1 Muscle Research Unit, Experimental and Clinical Research Center, a Cooperation between the Max Delbrück Center for Molecular Medicine in the Helmholtz Association and the Charité—Universitätsmedizin Berlin, 13125 Berlin, Germany

2 Max Delbrück Center for Molecular Medicine in the Helmholtz Association (MDC), 13125 Berlin, Germany.

3 Charité Universitätsmedizin Berlin, 13125 Berlin, Germany.

* Correspondence: haicui.wang215@gmail.com (H.W.); simone.spuler@charite.de (S.S.);

Tel.: +49-30-450-540-518 (H.W.); +49-30-450-540-501 (S.S.); Fax: +49-30-450-540-914 (H.W. and S.S.)

Supplementary FigureS1 Mutation impact on lamin proteins

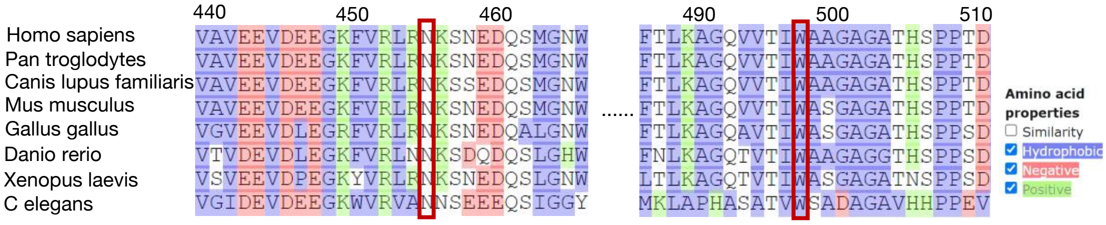

Sequence conservation of affected amino acid (N456, W498) across species. The canonical ones of lamin proteins were chosen from Uniprot and aligned in Uniprot.

Supplementary FigureS2 Generation of patient-derived iPSCs and quality control

A

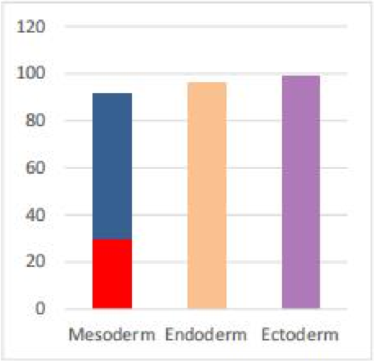

	Marker	% of cells	total
Mesoderm	CD140b	30.0	91.4
	CD144	61.4	
Endoderm	Sox17/CXCR4	96.5	
Ectoderm	Sox2/Pax6	98.7	

B

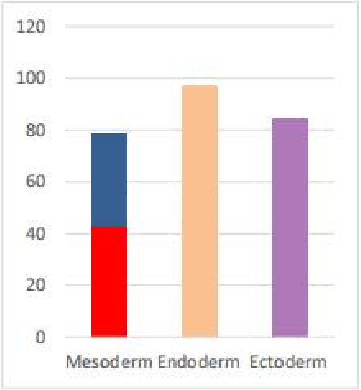

	Marker	% of cells	total
Mesoderm	CD140b	42.8	78.9
	CD144	36.1	
Endoderm	Sox17/CXCR4	96.9	
Ectoderm	Sox2/Pax6	84.6	

C

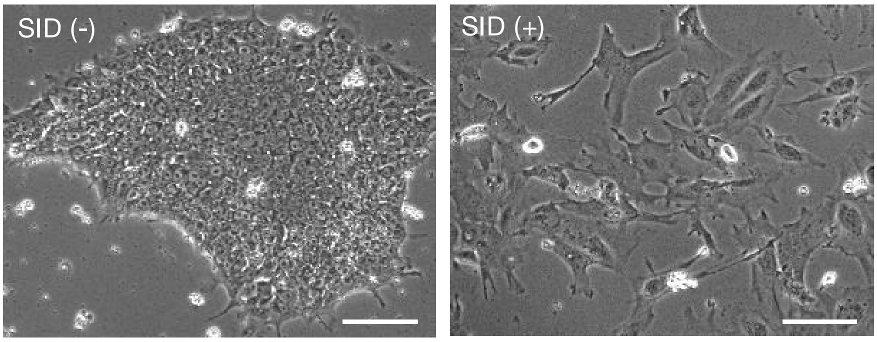

D

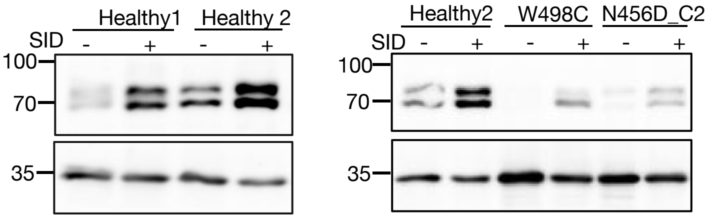

A-B. The cell line iPSC N456D_C1 (A) and N456D_C2 (B) shows potency to differentiate into mesoderm, ectoderm and endoderm lineages. N456D_C1 (A) or C2 (B): patient hiPSCs$^{LMNAc.1366A>G/LMNA_WT}$ clone 1 or clone 2.

Other characterization also performed but not showed here including:

-The lineage markers CD140b, CD144 (Mesoderm), Sox2, Pax6 (Ectoderm) and Sox17.

-CD184 (Endoderm) showed positive FACS results.

-The free of mycoplasma and sendai virus.

-The SNP karyotyping with PBMCs as control and also STR analysis.

C. The zoomed images of Figure 2B showed the the dissociated of the iPSC colony of the small rounded cells to enlarged, separated, adhensive cells. Scale bar 100 μm.

D. Western blot of laminA/C protein expression in healthy and patient-derived iPSCs prior to and after SID. A second healthy control iPSC line (healthy2) and a second clone for patient iPSC LMNA N456D (N456D_C2) were included. Healthy1: hiPSC$^{\text{LMNA_WT/LMNA_WT}}$; W498C: patient hiPSCs$^{\text{LMNAc.1494G>T/LMNA_WT}}$; N456D_C2: patient hiPSCs$^{\text{LMNAc.1366A>G/LMNA_WT}}$ clone 2.

Supplementary FigureS3 LMNA mRNA expression in patient-derived iPSCs

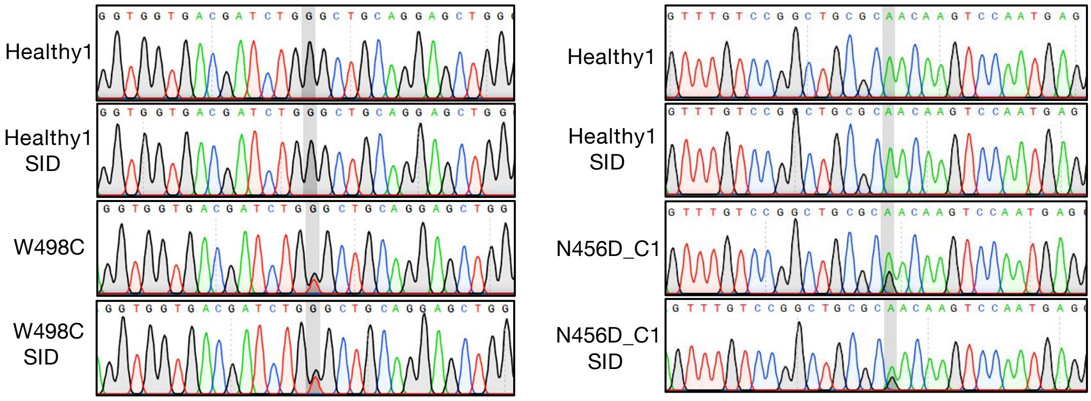

LMNA mRNA expression in healthy and patient-derived iPSCs prior to and after SID. Sequencing results for reverse transcription (RT) product from mRNAs confirmed the expression of both alleles in patient-derived iPSCs. Healthy1: hiPSC$^{\text{LMNA_WT/LMNA_WT}}$; W498C: patient hiPSCs$^{\text{LMNAc.1494G>T/LMNA_WT}}$; N456D_C1: patient hiPSCs$^{\text{LMNAc.1366A>G/LMNA_WT}}$ clone 1.

Supplementary FigureS4 LMNA co-regulated genes A B

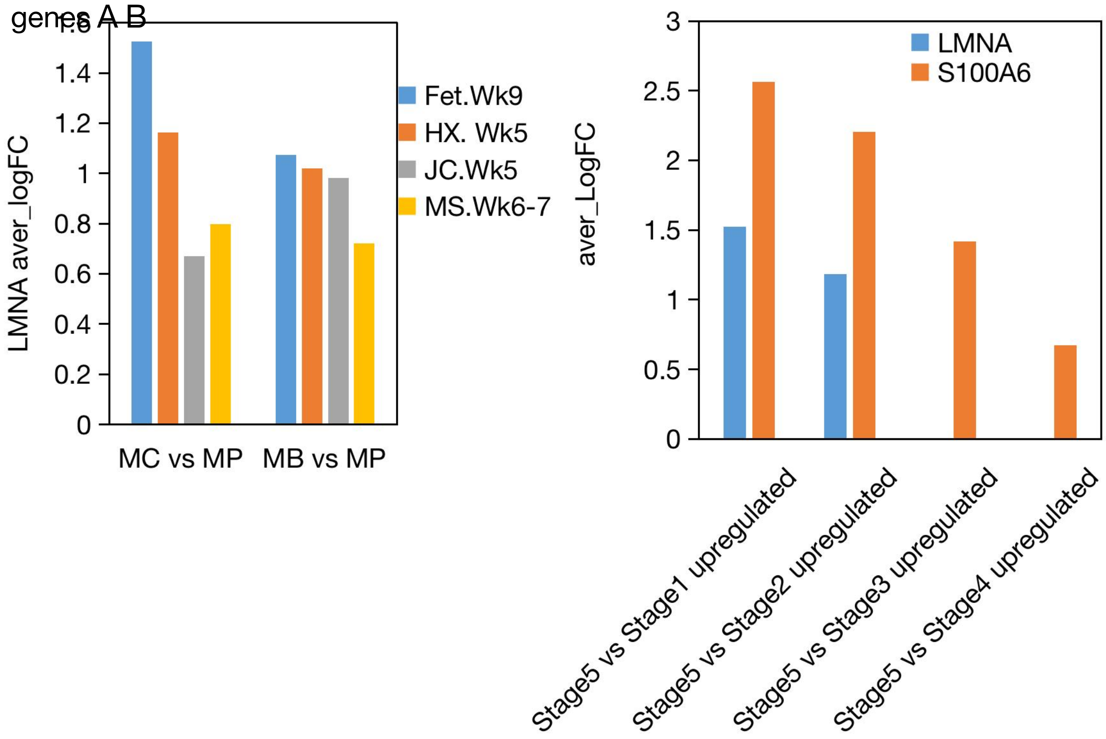

C

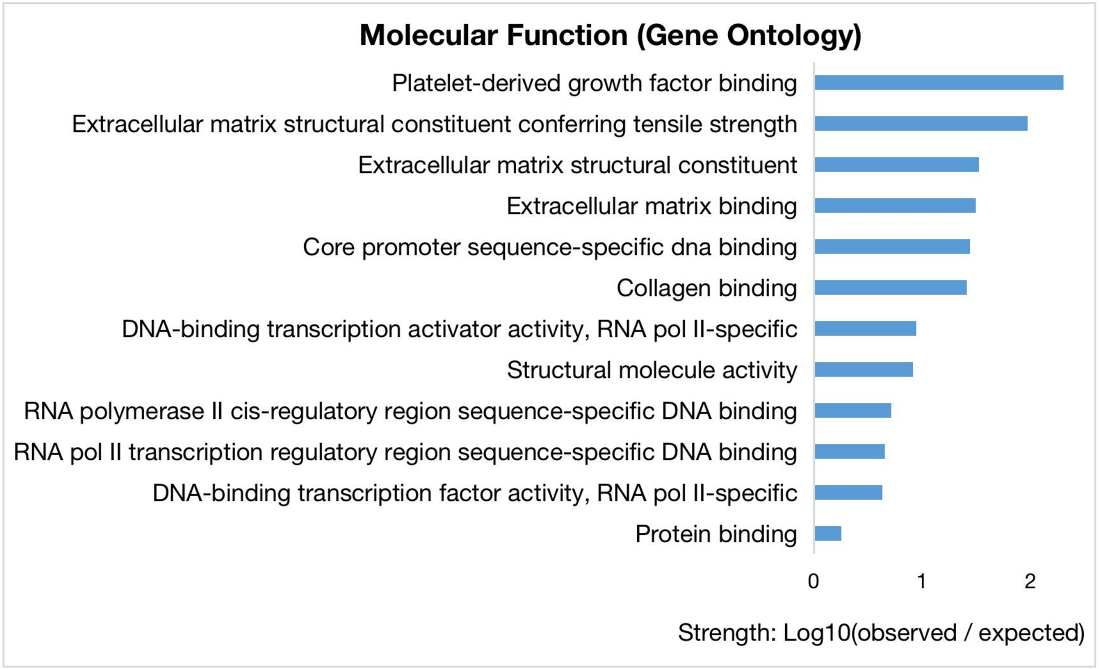

D

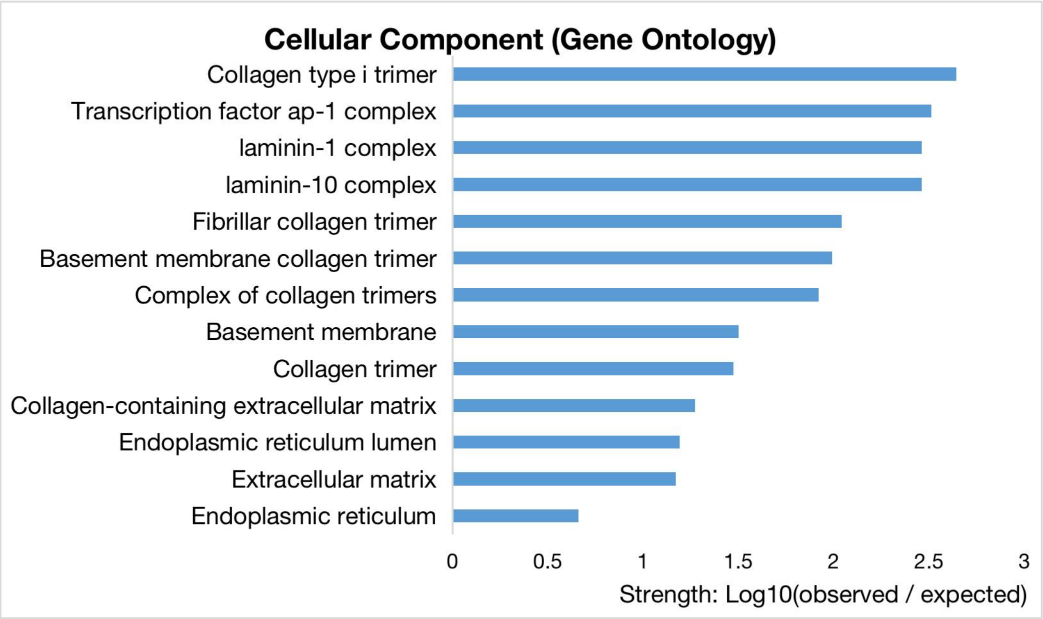

E

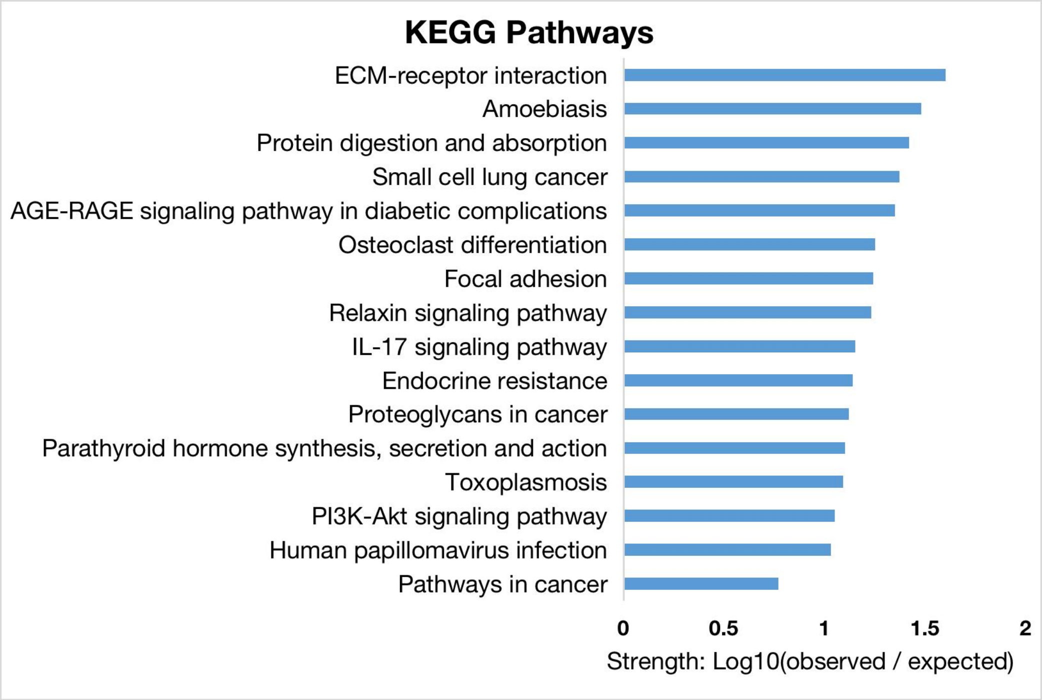

A. RNA seq data from Xi et al. 2020 ((13), https://www.ncbi.nlm.nih.gov/pmc/articles/PMC7367475/bin/NIHMS1590276-supplement-Table_S2.xlsx) showed LMNA gene was upregulated in myocytes (MC) and myoblasts (MB) compared to myogenic progenitors (MP) in early stages of three myogenic differentiation methods (HX/JC/MS) and in fetal muscle samples.
B. RNA seq data from Xi et al., 2020 ((13), https://www.ncbi.nlm.nih.gov/pmc/articles/PMC7367475/bin/NIHMS1590276-supplement-Table_S3.xlsx) showed co-regulated gene S100A6 and LMNA along the different myogenesis stages.
C-E. Gene ontology analysis and KEGG pathway analysis.

Supplementary FigureS5 LMNA co-regulated gene prior to and after SID

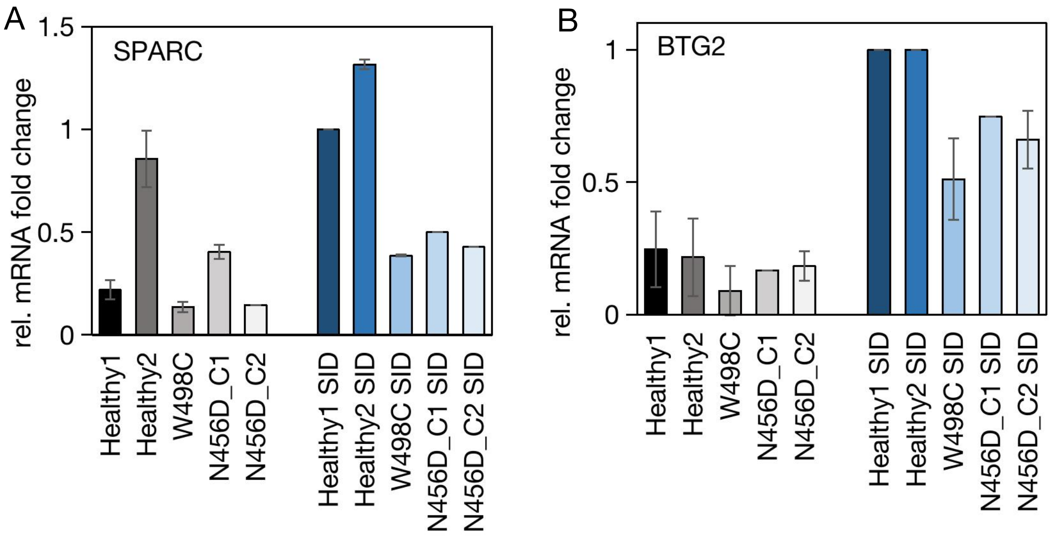

Gene expression of LMNA co-regulated genes SPARC (A) and BTG2 (B) prior to and after SID. Two healthy control iPSC cell line (healthy1 and healthy2) and two clones of iPSCs from LMNA N456D patients (N456D_C1, N456D_C2) were included in validating the selected gene expression. Healthy1 and Healthy2: hiPSC$^{LMNA_WT/LMNA_WT}$; W498C: patient hiPSCs$^{LMNAc.1494G>T/LMNA_WT}$; N456D_C1 or C2: patient hiPSCs$^{LMNAc.1366A>G/LMNA_WT}$ clone 1 or clone 2.

Supplementary FigureS6 CBE base editing with double vectors in patient–derived iPSCs

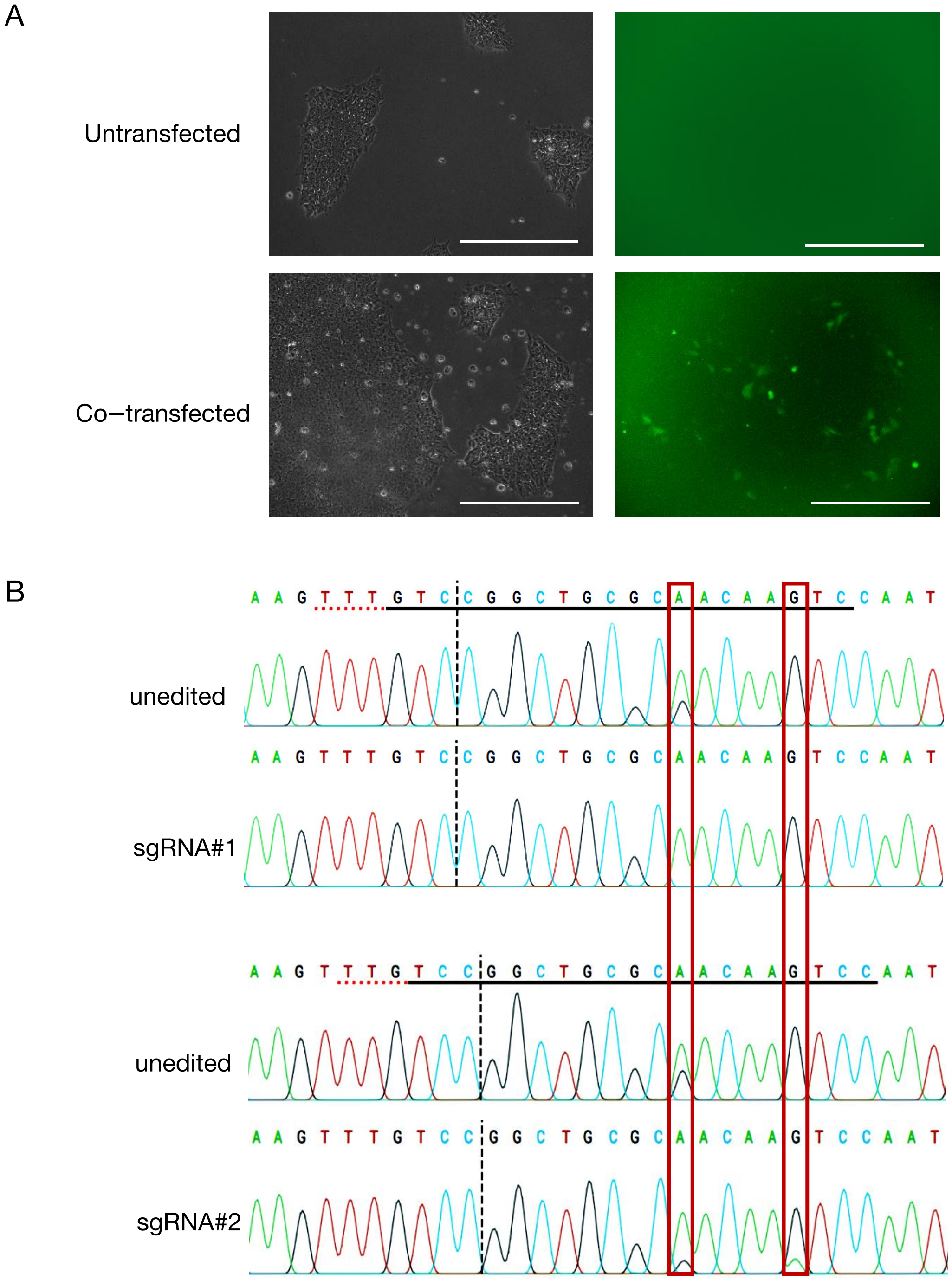

A. Co–transfection of one vector carrying the CBE4max_SpRY with a GFP reporter and one vector carrying the sgRNA to iPSCs N456D_C1 with lipofectamine. N456D_C1: patient hiPSCs$^{LMNAc.1366A>G/LMNA_WT}$ clone 1.

B. The iPSCs after 24 hour transfection. Positive GFP signal indicated the expression of the CBE4max_SpRY

C. Cells were FACS sorted after 36 hours of transfection and the genomic DNA sequence was analyzed. The sgRNA#2 introduced extra bystander editing.

Supplementary FigureS7 LMNA and its co-regulated gene expression after base editing

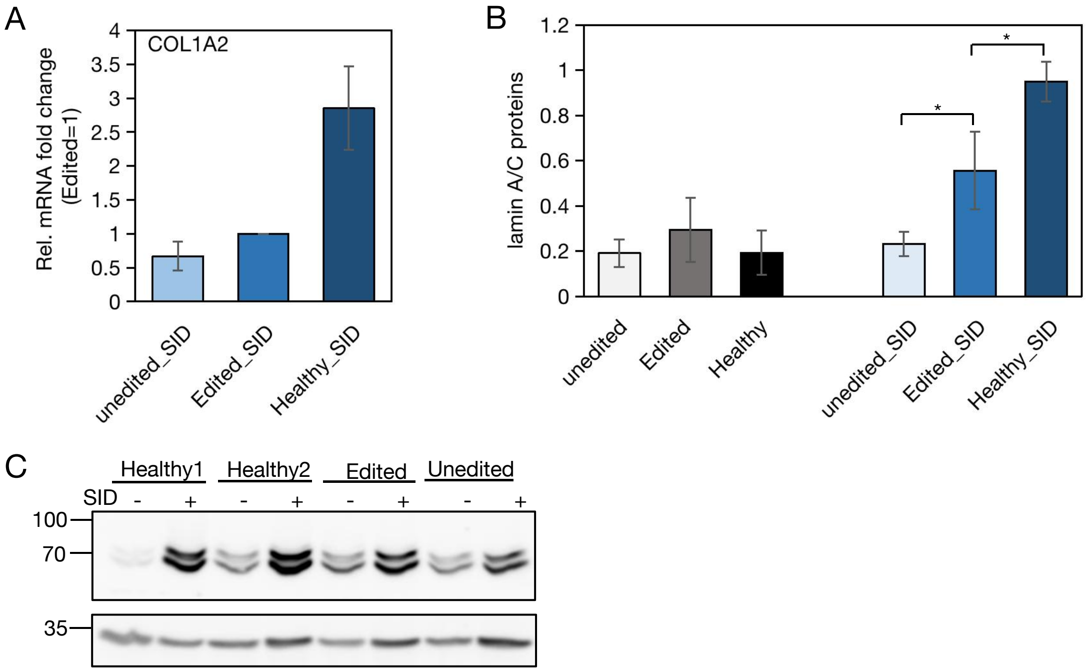

A. LMNA co-regulated gene COL1A2 mRNA expression in edited vs unedited iPSC N456D_C2 after SID (N=3; P<0.05). Healthy control includes both healthy1 and healthy2.

B-C. The lamin A/C protein levels between unedited and edited iPSC N456D_C2 iPSCs, between the edited N456D_C2 and the healthy control (N=3, P<0.05). Healthy control includes both healthy1 and healthy2. Healthy1 and Healthy2: hiPSC$^{LMNA_WT/LMNA_WT}$; N456D_C2: patient hiPSCs$^{LMNAc.1366A>G/LMNA_WT}$ clone 2.

Supplementary Table S1 Primers used for sequencing and qPCR

Primer Name	Applications	Sequence (5´ to 3´)
LMNA_P1_fw	genotyping N456D	GCAACTGGCCTTGACTAGAC
LMNA_P1 rev		GCTCAAGGTCTCACAGCCA
LMNA_P2_fw	genotyping W498C	CAGTGACAGGGGTGTGTGTAG
LMNA_P2 rev		CTTCCCCAGTGGAGTTGATG
LMNA_P3_fw	RT N456D	CTTCTGGACATCAAGCTGGC
LMNA_P3_rev		TTCTGTGCCTTCCACACCAG
LMNA_P3_fw	RT W498C	CTTCTGGACATCAAGCTGGC
LMNA_P4_rev		TCCATCCTCATCCTCGTCGT
LMNA_P5_fw	qPCR primers	CGCAGGCCAGCTCCAC
LMNA_P5_rev		TCCTGCAGGTCCTCCTTCTC
LMNA_P6_fw		GTGGAAGGCACAGAACACCT
LMNA_P4_rev		TCCATCCTCATCCTCGTCGT
FOSB fw		GCTGCAAGATCCCCTACGAAG
FOSB rev		ACGAAGAAGTGTACGAAGGGTT
FOS P1 fw		CTGTCAACGCGCAGGACTTC
FOS P1 rev		TCATGGTCTTCACAACGCCA
FOS P2 fw		CTGATACACTCCAAGCGGAGAC
FOS P2 rev		AGGGTCATTGAGGAGAGGCA
S100A6 fw		CTCCCTACCGCTCCAAGC
S100A6 rev		CTGGAAGTTCACCTCCTGGTC
PTRF P1 FW		GAGGACCCCACGCTCTATATT
PTRF P1 ReV		CCCCGATGATTTTGTCCAGGA
PTRF P2 FW		GGGCCGTAGACCAGATCCA
PTRF P2 ReV		CTTGCTCACCGTATTGCTCGT
LGALS1 FW		CTGTGCCTGCACTTCAACC
LGALS1 rev		CATCTGGCAGCTTGACGGT
COL3A1 P1 fw		GGAGCTGGCTACTTCTCGC
COL3A1 P1 rew		GGGAACATCCTCCTTCAACAG
COL3A1 P2 fw		GCCAAATATGTGTCTGTGACTCA
COL3A1 P2 rev		GGGCGAGTAGGAGCAGTTG
SPARCL1 p1 FW		ACAGGGCAAGAGTTCTAGCCA
SPARCL1 p1 rev		TTGGTGCATACTCCAAATTCACA
SPARCL1 p2 FW		CCAACTGAAGGTACATTGGACAT
SPARCL1 p2 rev		CTGTGAAGGAACTAACACCAGG
SPARC p1 FW		TGAGGTATCTGTGGGAGCTAATC
SPARC p1 rev		CCTTGCCGTGTTTGCAGTG
SPARC p2 FW		CCCATTGGCGAGTTTGAGAAG
SPARC p2 rev		CAAGGCCCGATGTAGTCCA
S100A4 fw		GATGAGCAACTTGGACAGCAA
S100A4 rev		CTGGGCTGCTTATCTGGGAAG

S100A10 fw		GGCTACTTAACAAAGGAGGACC
S100A10 rev		GAGGCCCGCAATTAGGGAAA
EGR1 fw		CCACGCCGAACACTGACATT
EGR1 rev		GAGGGGTTAGCGAAGGCTG
COL4A1 fw		GGACTACCTGGAACAAAAGGG
COL4A1 rev		GCCAAGTATCTCACCTGGATCA
LAMB1 fw		CACAAGCCCGAACCCTACTG
LAMB1 rev		GACCACATTTTCAATGAGATGGC
CD44 fw		CTGCCGCTTTGCAGGTGTA
CD44 rev		CATTGTGGGCAAGGTGCTATT
COL15A1 fw		CTGCCCTCGTCCGTATCCT
COL15A1 rev		CTGATGGCGAAGTCCCTGA
LAMA2 fw		TGCTGTCCTGAATCTTGCTTC
LAMA2 rev		AGCATTTGTAATCGGGTGTCTC
NR3C1 fw		TGCCGCTATCGAAAATGTCTT
NR3C1 rev		GGGTAGGGGTGAGTTGTGGT
COL1A2 fw		GGCCCTCAAGGTTTCCAAGG
COL1A2 rev		CACCCTGTGGTCCAACAACTC
P4HA2 fw		GGCCTGGTTTGGTGTCCTG
P4HA2 rev		GCCCAGCTCTTAATCTTGGAAAG
AHNAK fw		AACTCAAGGGTCCAAAGTTCAAG
AHNAK rev		GAGAGACATCCACATCACCTTTC
BTG2 fw		ACGGGAAGGGAACCGACAT
BTG2 rev		CAGTGGTGTTTGTAGTGCTCTG
COL6A1 fw		ACAGTGACGAGGTGGAGATCA
COL6A1 rev		GATAGCGCAGTCGGTGTAGG
JUNB fw		ACAAACTCCTGAAACCGAGCC
JUNB rev		CGAGCCCTGACCAGAAAAGTA
DLK1 fw		AGGGTCCCCTTTGTGACCA
DLK1 rev		GCAGGCCCGAACATCTCTATC
MATN2 fw		GATCCTCGGACAGATCGTCCT
MATN2 rev		CTGCCCGCTTGTTCTCACA
SPATS2L fw		AAGCAGCATCAAGGCAACAAA
SPATS2L rev		TTCTCGCAGCCATTCATGGG
CD82 fw		GCTCATTCGAGACTACAACAGC
CD82 rev		GTGACCTCAGGGCGATTCA
OLFML2A fw		CACGCCTACGTCCACAAGG
OLFML2A rev		TCATAGTGCCTCAACTGCTCA
CD63 fw		CAGTGGTCATCATCGCAGTG
CD63 rev		ATCGAAGCAGTGTGGTTGTTT
TXNIP fw		TGTGTGAAGTTACTCGTGTCAAA
TXNIP rev		GCAGGTACTCCGAAGTCTGT
SOGA3 fw		GTTCGCTGCGACAGACAATC

SOGA3 rev		GGCTCCCGGTCTTGTTACC
TPPP3 fw		AAGTCTGCTCGGGTCATCAAC
TPPP3 rev		GAGCCCGTGTATCTGCTGG
RASSF4 fw		TAAGACCTCCGTGTTTACTCCA
RASSF4 rev		GATGTAGAGTGCGAACTCACTG
SEMA3C fw		TTTGCGTGTTGGTTGGAGTAT
SEMA3C rev		TCCTGTAGTCTAAAGGATGGTGG

Supplementary Table S2 Gene correlation score with LMNA
(Extracted from Xi et al., 2020, RNA–seq data link:
https://www.ncbi.nlm.nih.gov/pmc/articles/PMC7367475/bin/NIHMS1590276-supplement-Table_S6.xlsx)

Target_Gene	Corr (to LMNA)
S100A6	0.227843323
PTRF (CAVIN1)	0.223155381
SPARCL1	0.222434649
DLK1	0.222315039
LGALS1	0.212318877
COL3A1	0.199294588
MATN2	0.196673838
SPATS2L	0.187344991
SPARC	0.181522567
S100A4	0.176378223
CD82	0.174338061
MEG3	0.173108788
OLFML2A	0.171756085
S100A10	0.171339814
EGR1	0.160695221
FOSB	0.158278831
COL4A1	0.157229474
CD63	0.154926244
LAMB1	0.153986923
TXNIP	0.151123669
CD44	0.148691777
COL15A1	0.147519494
LAMA2	0.1470055
NR3C1	0.145894724
COL1A2	0.136816817
P4HA2	0.134948929
FOS	0.134449557
SOGA3	0.1334006
AHNAK	0.132955064
TPPP3	0.132569713
BTG2	0.130908187
COL6A1	0.127789726
RASSF4	0.127020642
JUNB	0.126404984
SEMA3C	0.125539208

SupplementaryTableS3 LMNA co–regulated gene expression (fold change) prior to and after SID

Gene	Healthy1	Healthy1_SID	W498C	W498C _SID	N456D	N456D _SID
S100A6	1.00	74.26	0.26	5.71	1.29	17.62
COL3A1*	1.00	49.91	0.71	7.22	0.78	27.98
LGALS1	1.00	13.06	0.47	1.70	0.74	2.91
AHNAK	1.00	12.44	0.19	9.58	1.07	14.50
CD44	1.00	7.16	0.70	3.05	1.01	4.31
NR3C1	1.00	7.01	1.40	6.95	2.20	8.27
SEMA3C	1.00	6.70	0.30	0.71	0.61	3.95
OLFML2A	1.00	6.47	2.88	3.66	7.43	5.34
PTRF P1	1.00	6.12	0.05	0.56	0.56	5.85
LMNA	1.00	5.41	0.32	1.88	0.65	2.53
S100A10	1.00	5.20	0.35	1.26	0.53	2.64
COL1A2	1.00	4.83	0.38	1.27	1.71	2.79
P4HA2	1.00	4.49	1.72	2.42	4.62	3.46
S100A4	1.00	4.18	0.76	1.24	3.90	2.65
MATN2	1.00	4.01	0.43	1.16	2.03	3.00
SPARC	1.00	3.98	0.66	1.57	1.53	2.06
COL4A1	1.00	3.32	0.80	1.14	2.02	2.12
LAMB1	1.00	3.00	1.33	2.11	1.68	2.67
CD63	1.00	2.57	0.81	1.60	3.87	2.32
EGR1	1.00	2.46	5.47	8.28	1.73	3.97
BTG2	1.00	2.39	1.06	2.45	2.30	1.32
SPARCL1	1.00	1.83	1.61	1.12	3.67	2.12
FOSB	1.00	1.80	1.80	1.03	1.96	1.78
SPATS2L	1.00	1.78	0.70	0.83	0.95	1.81
DLK1	1.00	1.68	0.67	2.84	15.22	2.64
TXNIP	1.00	1.67	2.10	1.28	0.76	3.65
FOS	1.00	1.64	1.02	0.88	0.77	1.22
LAMA2	1.00	1.46	0.70	0.68	2.06	1.13
CD82	1.00	1.35	1.33	1.21	4.54	1.25
COL6A1	1.00	1.26	0.83	0.47	2.64	1.24
TPPP3	1.00	0.79	1.00	0.88	2.27	0.86
JUNB	1.00	0.60	0.85	0.52	1.56	1.00
SOGA3	1.00	0.59	1.47	0.29	1.20	0.37
RASSF4	1.00	0.46	1.43	0.77	2.28	0.67
COL15A1	1.00	0.31	0.64	0.23	1.12	0.33

(*COL3A1 very low expression with Ct>28 in all samples, excluded)
